# Effects of Whole-Body Vibration and Balance Training on Female Athletes with Chronic Ankle Instability

**DOI:** 10.3390/jcm10112380

**Published:** 2021-05-28

**Authors:** Wen-Dien Chang, Shuya Chen, Yung-An Tsou

**Affiliations:** 1 Department of Sport Performance, National Taiwan University of Sport, Taichung 404401, Taiwan; changwendien@ntus.edu.tw; 2 Department of Physical Therapy and Graduate Institute of Rehabilitation Science, China Medical University, Taichung 40402, Taiwan; sychen@mail.cmu.edu.tw; 3 Department of Otolaryngology-Head and Neck Surgery, China Medical University Hospital, Taichung 40402, Taiwan; 4 Department of Audiology and Speech-Language Pathology, Asia University, Taichung 41354, Taiwan

**Keywords:** chronic ankle instability, whole-body vibration, balance training

## Abstract

We explored the effects of 6-week whole-body vibration (WBV) and balance training programs on female athletes with chronic ankle instability (CAI). This randomized controlled study involved female athletes with dominant-leg CAI. The participants were randomly divided into three groups: WBV training (Group A), balance training (Group B), and nontraining (control group; Group C). Groups A and B performed three exercise movements (double-leg stance, one-legged stance, and tandem stance) in 6-week training programs by using a vibration platform and balance ball, respectively. The Star Excursion Balance Test (SEBT), a joint position sense test, and an isokinetic strength test were conducted. In total, 63 female athletes with dominant-leg CAI were divided into three study groups (all *n* = 21). All of them completed the study. We observed time-by-group interactions in the SEBT (*p* = 0.001) and isokinetic strength test at 30°/s of concentric contraction (CON) of ankle inversion (*p* = 0.04). Compared with the control group, participants of the two exercise training programs improved in dynamic balance, active repositioning, and 30°/s of CON and eccentric contraction of the ankle invertor in the SEBT, joint position sense test, and isokinetic strength test, respectively. Furthermore, the effect sizes for the assessed outcomes in Groups A and B ranged from very small to small. Female athletes who participated in 6-week training programs incorporating a vibration platform or balance ball exhibited very small or small effect sizes for CAI in the SEBT, joint position sense test, and isokinetic strength test. No differences were observed in the variables between the two exercise training programs.

## 1. Introduction

Lateral ankle sprain is one of the most common sports injuries and often causes a decrease in neuromuscular control and loss of proprioception [1]. Neuromuscular control helps maintain the functional stability of the ankle, whereas proprioception influences ankle joint position and sense of movement. Impairments in these functions cause reduced dynamic balance and reaction time of the ankle joint during exercise [2]. An ankle sprain resulting in damage to the lateral ankle capsuloligamentous complex can cause sequelae, such as recurring ankle sprains. It decreases ankle function as well as causing a loose feeling, and this postinjury symptom is classified as chronic ankle instability (CAI) [3]. Athletes with CAI may experience limited functionality of their lower extremities, which may affect their sports performance [4]. Therefore, specific balance exercises for athletes with CAI are critical in rehabilitation and training programs and could effectively reduce the risk of ankle sprain during sports activities.

Balance training is a progressive type of exercise performed on an unstable surface, and the resultant efferent output causes changes in α motor neuron excitability [5]. Balance training was used to improve muscle excitability in the ankle joint and increase motor control for CAI [6]. Whole-body vibration (WBV) training is another popular method used in CAI rehabilitation [7]. WBV training was performed on an oscillating vibration platform, which activates muscle spindles to facilitate tonic vibration reflex [8]. This training also enhances α motor neuron excitability and the synchronization of motor units to increase motor control in the ankle [9]. To the best of our knowledge, there have only been a few studies comparing the effects between WBC and traditional balance training on CAI. Therefore, WBV and balance training programs were designed and used for athletes with CAI in the current study.

For basketball and volleyball players, ankle stability and motor control are crucial for ground impact during jumping and landing. The dominant leg is a commonly discussed factor in these types of actions by injured athletes because it plays a critical role in object manipulation and lead-out movements [10]. However, the nondominant leg performs a stabilizing and supporting role in sports activities [10]. Therefore, the dominant leg is highly susceptible to sports injuries such as ankle sprain and may suddenly become injured during jumping and landing on an unstable surface. A study by van Melick et al. revealed that female athletes were more likely to jump using the dominant leg than male athletes [11]. A systematic review revealed that female athletes sustained ankle sprains more often than male athletes [12]. Sex-related differences in the epidemiology of ankle injuries were noted in sports injury protection [12]. Ristolainen et al. indicated female athletes tend to have a higher risk of sport-related ankle injury than male athletes [13]. Therefore, importance needs to be placed on an effective strategy of rehabilitation for female athletes. Previous studies have also reported a high incidence of ankle injuries in basketball and volleyball players [14,15]. Therefore, investigating the effects of specific interventions for CAI in female basketball and volleyball athletes is essential. Our study aim was to compare the effects of 6-week WBV and balance training on dominant-leg CAI in female athletes.

## 2. Methods

This study was a randomized controlled trial approved by the Institutional Review Board of China Medical University Hospital (CRREC-106-063). Participants with CAI were recruited from women’s basketball and volleyball teams at neighboring colleges. For inclusion criteria, female athletes had to have a history of at least one ankle sprain, lateral ankle instability of the dominant leg with a severity score ≤24 measured using the Cumberland Ankle Instability Tool, and a continual feeling of the ankle ‘‘giving way’’ after one year [16]. The dominant leg was used for lead-out movements and was determined as the foot used to kick a ball [10]. Exclusion criteria were acute ankle sprain, a history of surgery in both legs, and any musculoskeletal diseases of the lower extremities. The sample size calculation using G*Power software reported by Sefton et al. [17] was used, which resulted in a total of 21 participants. We also tried to use the statistical power of 80%, α level of 0.05, and effect size (f = 0.25) to calculate via G*Power software (version 3.1.9.2; Heinrich-Heine-Universität, Düsseldorf, Germany). A total sample size of 46 was calculated and required. In the current study, the estimated sample size was set to at least 48 participants (16 participants per group), which was a statistically adequate sample size.

### 2.1. Study Procedures

**Figure 1:**
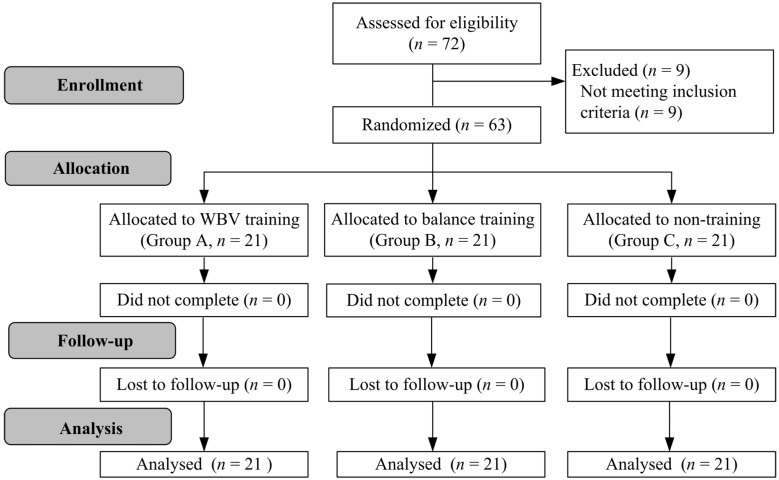
Flowchart of the current study. WBV: whole-body vibration.

This experimental trial was conducted at the end of the semester, and there was no practice or competition during this study period. The participants were randomly divided into three groups: Group A, who completed a 6-week WVB training program; Group B, who completed a 6-week balance training program; and Group C, who did not participate in a training program (Figure 1). All participants continued their normal daily activity and were instructed not to receive any treatments or therapy for CAI. The participants were assessed before and after the study. The participants underwent three assessments, the Star Excursion Balance Test (SEBT), a joint position sense test, and an isokinetic strength test, consecutively, and their performance was evaluated by the same physiotherapist. The participants and researchers were not blinded to the study process.

### 2.2. Exercise Training Program

**Figure 2:**
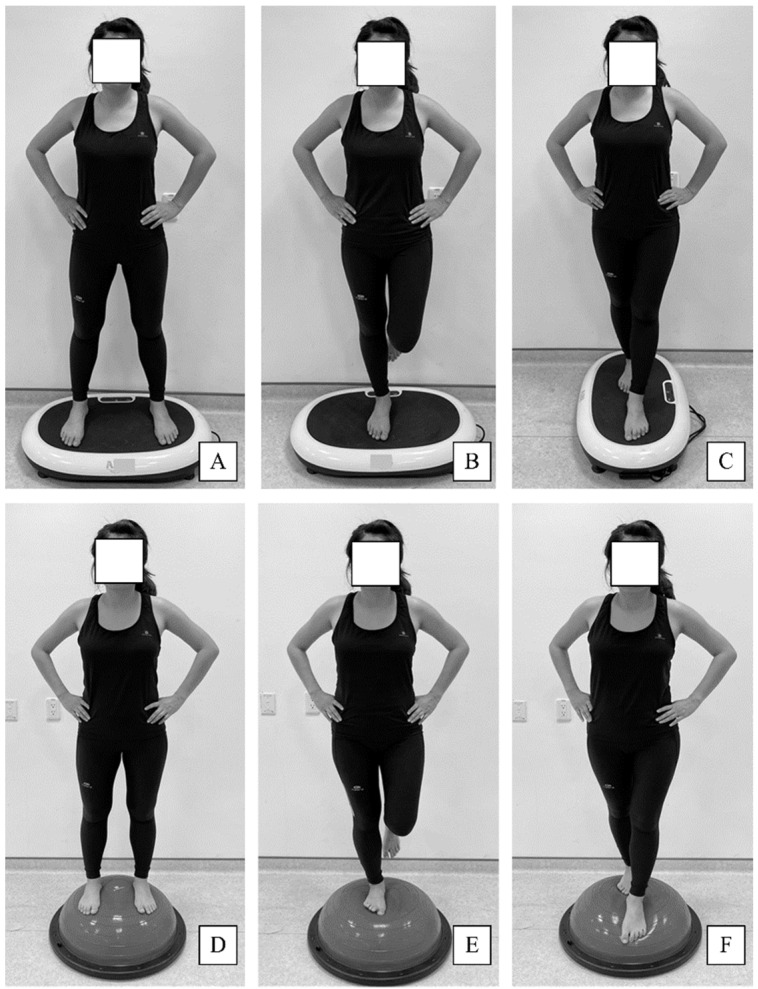
Demonstration of exercises on the vibration platform (**A**–**C**) and balance ball (**D**–**F**). (**A**,**D**): double-leg stance; (**B**,**E**): one-legged stance; (**C**,**F**): tandem stance.

Groups A and B performed the same exercises during the 6-week training programs, but Group A used a vibration platform (AIBI Power Shaper, AIBI Fitness, Singapore), and Group B used a balance ball (BOSU Balance Trainer, Fitness Quest, Ashland, OH, USA). Group A performed the exercises while standing on the vibration platform, which operated at a frequency and amplitude of 5 Hz and 3 mm, respectively. Group B participants performed the exercises on the balance ball. Participants in both groups were asked to maintain balance on either leg or an affected leg while having eyes closed. Both training programs were conducted respectively three times per week for 6 weeks and consisted of a 5-min warm-up exercise, a 20-min main exercise, and a 5-min cool-down exercise. They were designed with standard exercise prescription and clinical experience by one physical therapist. The main exercise comprised three exercise movements: a double-leg stance, a one-legged stance, and a tandem stance (Figure 2). Weeks 1–3 consisted of four sets of 45-s exercises with a 40-s rest interval between exercises, and weeks 4–6 consisted of five sets of 45-s exercises with a 30-s rest interval between exercises. The training programs in Group A and B were identical in the current study, except standing on different training devices. Group C participants were encouraged to continue their normal daily activity and avoid additional training programs or therapeutic exercise.

### 2.3. Assessments

#### 2.3.1. Star Excursion Balance Test

The SEBT was used to measure dynamic balance. The SEBT exhibited moderate-to-favorable interrater reliability (intraclass correlation coefficients [ICC] = 0.67–0.97) in an assessment of ankle instability [18]. An asterisk comprising eight tape segments joined at the center was placed on the floor. The tape segments were extended in eight directions (i.e., anterior, anterolateral, anteromedial, posteromedial, posterior, posterolateral, medial, and lateral) from the center at 45° angles [19]. The participants were asked to stand at the center of the asterisk using the leg with the involved ankle and then lightly touch the asterisk with the contralateral leg as far as possible in a direction chosen by the physiotherapist. The participants were instructed to maintain their balance in the one-legged stance by using the leg with the involved ankle and then return the contralateral leg to its initial position. Reach distances in the eight directions were recorded following three consecutive tests. The length of the involved leg was measured from the anterior superior iliac spine to the medial malleolus. The average reach distances in each direction were normalized according to the length of the involved leg and represented as a percentage.

#### 2.3.2. Joint Position Sense Test

The SYSTEM 3 PRO dynamometer (Biodex Medical Systems, Shirley, NY, USA) was used to conduct a joint position sense test measuring active and passive repositioning. The dynamometer exhibited medium-to-high reliability in an assessment of joint kinesthesia ability (r = 0.6–0.8) [20]. The participants laid in a supine position while blindfolded, and the involved ankle was placed on the ankle inversion-eversion footplate of the dynamometer with a plantar flexion of 15°. Three reference angles were established for ankle inversion, neutral ankle position, and ankle eversion (15°, 0°, and 10°, respectively), and the participants were asked to actively and passively reproduce the angle. The absolute value of the joint angle error represents the actual difference between the reference angle and the matching angle [21].

In active repositioning, the involved ankle was first placed in a neutral position and then moved to the inversion or eversion reference angle for 10 s. The participants were asked to actively reproduce the angle three times; the corresponding angles produced by the participants were then recorded. In passive repositioning, the involved ankle was also first placed in a neutral position and then moved to the inversion or eversion reference angle for 10 s. The involved ankle was then passively inverted and everted through a full range of motion by using 5°/s of angular velocity and stopped at the original reference angle by using a hand-held switch. Three passive repositioning trials were completed to reproduce the reference angle, and the corresponding angles were recorded. The average absolute values of the joint angle errors were subsequently analyzed.

#### 2.3.3. Isokinetic Strength Test

The SYSTEM 3 PRO dynamometer was also used to conduct the isokinetic strength test. The dynamometer exhibited high reliability (ICC coefficients = 0.87–0.96) and was effective in measuring the isokinetic strength of ankle joints [22]. Ankle invertor and evertor muscle strength was measured in terms of concentric contraction (CON) and eccentric contraction (ECC) at velocities of 30°/s and 120°/s, respectively [23]. Prior to testing, the participants warmed up for 10 min using general range-of-motion exercises for ankle joints. The participants sat in a chair with the backrest at a seatback tilt of 70°, and their trunks and pelvises were fixed with straps. The involved leg was fixed with a strap, and the involved foot was secured to ankle attachments with two straps. The tested ankle was positioned with 20° of plantar flexion, and the rotational axis of the dynamometer was leveled at the subtalar joint. Three repetitions were performed at velocities of 30°/s and 120°/s with 1-min rest intervals between each repetition [24]. The CON and ECC of the ankle inversion and eversion were calculated as peak torque normalized according to body weight. The respective ratios of inversion and eversion for ECC or CON at 30°/s and 120°/s were also calculated.

### 2.4. Statistical Analysis

Statistical analysis was performed using SPSS version 25.0 (SPSS, Chicago, IL, USA). The Shapiro–Wilk test was used to verify the normality of the data to ensure the normal distribution of all assessed variables (*p* > 0.05). Descriptive statistics were used, and all data are presented as the mean ± standard deviation. An analysis of variance (ANOVA) and chi-squared test were used for continuous and categorical variables, respectively, to compare the differences within groups. The results of the SEBT, joint position sense test, and isokinetic strength test were analyzed using a two-way repeated-measures ANOVA (three groups × two times) followed by a Bonferroni post hoc test. Effect size (d) was classified according to the scale of Cohen [25] into very small (<0.2), small (0.2–0.5), medium (0.5–0.8), and large (>0.8), and the effects of Groups A and B were determined to respectively compare Group C. Multivariable linear regression analysis was used to evaluate the association of main outcome measurements between the two training groups and the non-training group. Multivariable modelling was performed with R^2^ and β coefficients and specified as the change values of assessment variables for the prediction. The α level for all statistical analyses was set at 0.05.

## 3. Results

Sixty-three female athletes (50 basketball players and 13 volleyball players) with dominant-leg CAI participated following the inclusion criteria in our study. The participants were randomly divided using a random number generator into Groups A, B, and C (all *n* = 21, Figure 1). After the study process, no participants dropped out, and no participants reported adverse reactions. All of the participants completed the study. The demographics are presented in Table 1, and no significant differences were observed within the three groups (all *p* > 0.05).

The ANOVA outcomes are summarized in Table 2. In the results of multivariable linear regression (Table 3), the R^2^ values were 0.80 and 0.93 in Group A and B, respectively. Significant relationships between the change in values in active repositioning and 30°/s of ECC ankle inversion were noted (*p* < 0.05). Results of the SEBT among the three groups are presented in Table 4. To calculate the composite score of SEBT, the main effects of group (F_(2, 60)_ = 5.30, *p* = 0.03), time (F_(2, 60)_ = 67.78, *p* = 0.001), and time × group (F_(2, 60)_ = 17.84, *p* = 0.001) were observed. Post hoc tests indicated no significant differences in the SEBT within the groups before assessment in terms of composite score and individual directions (*p* > 0.05). Between Groups A and C, the anteromedial (*p* = 0.01, effect size: d = 1.25, 95% CI = 0.59–1.91), posterolateral (*p* = 0.03, effect size: d = 1.05, 95% CI = 0.41–1.70), and lateral (*p* = 0.03, effect size: d = 1.09, 95% CI = 0.44–1.74) directions in the SEBT were significantly different. However, no significant difference was observed in composite scores on the SEBT within the two groups (*p* > 0.05). Moreover, the results indicated that the SEBT composite score and individual directions were higher in Group B than in Group C (all *p* < 0.05). Within the two groups, a small effect size in composite score on the SEBT was observed (d = 2.34, 95% CI = 1.55–3.12), and very small to small effect sizes were observed for all individual directions (anterior, d = 1.70, 95% CI = 1.00–2.41; anterolateral, d = 1.53, 95% CI = 0.84–2.22; anteromedial, d = 1.88, 95% CI = 1.15–2.60; posteromedial, d = 1.21, 95% CI = 0.55–1.86; posterior, d = 1.74, 95% CI = 1.03–2.45; posterolateral, d = 2.13, 95% CI = 1.38–2.89; medial, d = 1.32, 95% CI = 0.65–1.99; and lateral, d = 2.18, 95% CI = 1.41–2.94).

The changes in active and passive repositioning data before and after assessment for the three groups are presented in Table 5. During active repositioning, for an ankle inversion of 15°, the main effects of group (F_(2, 60)_ = 3.35, *p* = 0.08), time (F_(2, 60)_ = 12.37, *p* = 0.006), and time × group (F_(2, 60)_ = 0.48, *p* = 0.63) were observed. For the neutral ankle position, the main effects of group (F_(2, 60)_ = 6.59, *p* = 0.01), time (F_(2, 60)_ = 12.26, *p* = 0.006), and time × group (F_(2, 60)_ = 1.58, *p* = 0.25) were observed. For an ankle eversion of 10°, the main effects of group (F_(2, 60)_ = 0.51, *p* = 0.61), time (F_(2, 60)_ = 8.27, *p* = 0.01), and time × group (F_(2, 60)_ = 1.60, *p* = 0.25) were observed. During passive repositioning, for an ankle inversion of 15°, neutral ankle position, and an ankle eversion of 10°, no statistical significance was observed in the main effects of group, time, and time × group (*p* > 0.05).

For active repositioning data, the within-group analysis of Group A revealed a significant decrease in neutral ankle position (*p* = 0.04) but no significant decreases for an ankle inversion of 15° (*p* = 0.16) and an ankle eversion of 10° (*p* = 0.13). In Group B, significant decreases for an ankle inversion of 15° (*p* = 0.01), neutral ankle position (*p* = 0.02), and an ankle eversion of 10° (*p* = 0.01) were observed. Post hoc tests indicated no significant differences within the three groups before assessment. Compared with Group C, Groups A and B exhibited significant decreases for an ankle inversion of 15°, neutral ankle position, and an ankle eversion of 10° (*p* < 0.05). Very small to small effect sizes were observed for an ankle inversion of 15° (d = −0.97, 95% CI = −1.61 to −0.33), neutral ankle position (d = −2.18, 95% CI = −2.94 to −1.41), and an ankle eversion of 10° (d = −0.95, 95% CI = −1.59 to −0.31) after assessment between Groups A and C. Between Groups A and C, small to medium effect sizes were also observed for an ankle inversion of 15° (d = −0.90, 95% CI = −1.54 to −0.27), neutral ankle position (d = −2.20, 95% CI = −2.97 to −1.43), and an ankle eversion of 10° (d = −0.89, 95% CI = −1.52 to −0.25) after assessment.

The CON and ECC data before and after assessment for the three groups are presented in Table 6. For 30°/s of CON ankle inversion, the main effects of group (F_(2, 60)_ = 3.01, *p* = 0.10), time (F_(2, 60)_ = 0.04, *p* = 0.82), and time × group (F_(2, 60)_ = 4.49, *p* = 0.04) were observed. For 30°/s of ECC ankle inversion, the main effects of group (F_(2, 60)_ = 14.02, *p* = 0.002), time (F_(2, 60)_ = 5.56, *p* = 0.04), and time × group (F_(2, 60)_ = 0.71, *p* = 0.51) were observed. Post hoc tests indicated no significant differences within the three groups for 30°/s of CON and ECC ankle inversion before assessment, but the number of ankle invertor muscle contractions in Group C was significantly lower than those in Groups A and B for 30°/s of CON (*p* = 0.01 and *p* = 0.02, respectively) and 30°/s of ECC (*p* = 0.01 and *p* = 0.001, respectively). Very small effect sizes were observed in 30°/s of CON ankle inversion after assessment in Group A (d = 1.24, 95% CI = 0.58–1.90) and Group B (d = 1.13, 95% CI = 0.47–1.78) compared with Group C. Small effect sizes were also observed in 30°/s of ECC ankle inversion after assessment in Group A (d = 1.15, 95% CI = 0.49–1.80) and Group B (d = 1.37, 95% CI = 0.69–2.04) compared with Group C. However, no significant main effects of group, time, and time × group (*p* > 0.05) were observed in 30°/s of ECC and CON ankle eversion. In addition, for 120°/s of CON and ECC ankle inversion or eversion, no statistical significance was observed in the main effects of group, time, and time × group (*p* > 0.05). Regarding the analysis of the ratios of inversion and eversion for ECC or CON at 30°/s and 120°/s, no isokinetic parameters were statistically significant after using two-way repeated-measures ANOVA (*p* > 0.05).

## 4. Discussion

Compared with the control group, the female athletes with dominant-leg CAI improved in the SEBT, the active repositioning portion of the joint position sense test, and 30°/s of CON and ECC of the ankle invertor in the isokinetic strength test after completing a 6-week WVB or balance training program. However, very small to small effect sizes for the assessed outcomes were observed between the exercise programs and control groups.

Sixty-three female athletes (50 basketball players and 13 volleyball players) with CAI participated in the study. Some prospective studies have reported that 19%–20% of ankle sprain rates occur in female basketball players [26,27]. Compared with male athletes, female athletes often experience greater laxity in the ankle joint and its ligaments. These sex-specific differences are caused by variations in hormone levels [28]. In addition, because the dominant leg is preferentially used for jumping and landing in basketball and volleyball, 80% of CAI in the dominant ankle is due to participation in sports [26]. Balance exercise programs have been designed specifically for female athletes, and the resulting improvements in balance and stability after completing such programs could reduce the risk of contact ankle sprains [29]. To our knowledge, the current study is the first to compare two exercise training programs with a control group. After 6 weeks, the WVB training group performed better than the balance training group did, and both groups exhibited improvements in dynamic balance, joint position sense, and isokinetic muscle strength after assessment. However, compared with the control group, both groups contributed to very small or small effect sizes of the assessed variables.

Balance deficit is the main cause of increased risk of recurring ankle sprain [30]. The exercise training programs, i.e., WBV and balance training, also play a decisive role in the rehabilitation process after an ankle sprain [31,32]. Some studies have suggested that balance training, especially by using the BOSU Balance Trainer, effectively reduces the balance deficit in patients with CAI [33,34]. WBV training is a popular method used for CAI rehabilitation and mitigating ankle instability. WBV training can increase α and γ neuron excitability and improve muscle spindle sensitivity, resulting in decreased muscular reaction time [8,9]. Therefore, the effects of WBV training on CAI could enhance ankle posture control during the SEBT, a dynamic balance measurement tool. Compared with the control group, female athletes with CAI experienced enhanced dynamic balance through WBV training and by using the BOSU Balance Trainer, both of which resulted in similar effects. However, both training programs contributed to very small or small effect sizes for CAI. Rendos et al. observed that WBV training does not cause acute dynamic balance improvements in patients with CAI [35]. Sierra-Guzmán et al. observed that compared with a control group, athletes with CAI exhibited moderate effect sizes (d = 0.54) in a composite score of SEBT after 6-week programs combining WBV training and the BOSU Balance Trainer [36]. Cloak et al. also observed a significant increase in the SEBT and revealed that 6-week programs combining WBV and a wobble board were effective for athletes with CAI [7]. Established programs combined with WBV may be a potential rehabilitation strategy for athletes with CAI, and should be studied in the future.

The joint position sense test measures joint kinesthesia and is used to measure the sensorimotor deficit of CAI [37]. Compared with the control group, athletes who underwent 6-week programs incorporating WBV training and the BOSU Balance Trainer showed improvement in the active repositioning of joint position sense at an ankle inversion of 15°, neutral ankle position, and an ankle eversion of 10°, but no significant improvement in passive repositioning (*p* > 0.05). Sousa et al. indicated that CAI could cause a decrease in joint position sense during ankle inversion, consequently affecting functional ankle movement [38]. In the current study, the reduced joint position error in ankle inversion and eversion is the contraction that was improved by both balance training programs. During active repositioning, the detection of movement sensation is markedly enhanced by ankle muscle contraction. We observed that the enhancement in active repositioning was better than that in passive repositioning because of the increase in spindle afferent activity and muscle strength [39]. Otzel et al. applied WBV at 35 Hz for CAI rehabilitation and reported no improvement in joint position accuracy or proprioception [40]. In the current study, the WBV training with a frequency of 5 Hz could improve the joint kinesthesia ability of ankle inversion and eversion in active repositioning for CAI, and the effects were the same as the balance training program. Baumbach et al. indicated that the frequency of WBV may be a key factor in CAI rehabilitation [41]. A frequency of <10 Hz is used for relaxing muscles; a frequency between 10 Hz and 20 Hz is used for coordination exercises, and a frequency >20 Hz is used for enhancing muscle contractions in WBV training [42]. However, the effects of various WBV frequencies on joint position sense in CAI are unknown, and additional research on this issue is warranted.

Regarding ankle strength, Wilkerson et al. reported significantly greater deficits in inversion strength than in eversion strength by using an isokinetic dynamometer [43]. Concentric invertor strength deficits are commonly found in patients with CAI, resulting in deep peroneal nerve dysfunction or selective neuromuscular inhibition after ankle sprain [44]. Ko et al. analyzed isokinetic ankle invertor and evertor muscle strength at angular velocities of 30°/s and 120°/s in patients with CAI and observed a severe invertor strength deficit at an angular velocity of 30°/s [45]. In our findings, the ECC and CON invertor strengths at 30°/s of angular velocity in the WBV and balance training group were significantly higher than those in the control group (*p* < 0.05). However, no significant differences in ECC and CON at angular velocities of 30°/s and 120°/s were observed in both groups before and after assessment (*p* > 0.05). Three exercise movements (double-leg stance, one-legged stance, and tandem stance) were applied in our training program, which consisted of 6-week programs incorporating static balance training exercises on a vibration platform or balance ball. During the exercises, the athletes (who had CAI) were instructed to maintain their balance on an unstable surface. The low frequency of 5 Hz of WBV in addition to static posture control exercises improved the ankle invertor strengths in ECC and CON at an angular velocity of 30°/s. This strategy can be used as ankle invertor strength training for mitigating strength deficits in patients with CAI. The resultant increase in invertor isokinetic strength at a low angular velocity could be applied to reduce the capacity of control lateral postural sway during weight-bearing activities and to alleviate CAI symptoms [46].

Some studies have suggested that patients with CAI possess limited evertor isokinetic strength in injured ankles [47,48] and that an increase in evertor strength could assist the lateral ligaments in supporting a sudden ankle inversion movement [49]. The ECC of eversion/CON of inversion strength ratio, which is a functional agonist/antagonist strength ratio, is focused on outcome measurements for patients with CAI. Because normal movement patterns of lower extremities and gait patterns involve the interaction of agonist and antagonist strength, the strength ratio was used as an indicator of rehabilitation training for patients with CAI [50]. Brent et al. used the ankle eversion/inversion strength ratio to assess the outcomes of a 6-week strength training program for CAI and indicated that the balance of ankle eversion and inversion strength can support injured ligaments and improve ankle stability [49]. In the current study, the ankle eversion isokinetic strength and eversion/inversion isokinetic strength ratio at 30°/s and 120°/s angular velocities and the ECC of eversion/COM of inversion strength ratio did not exhibit significant differences within and between the groups (*p* > 0.05). Brent et al. reported that ankle invertor and evertor strength was significantly increased in college students with CAI after a 6-week strength training program [49]. Mohd Salim et al. also observed that the ankle eversion/inversion strength ratio improved in the ankles of patients with CAI after a 1-week standard physiotherapy program [51]. These studies suggest that ankle eversion or inversion strength in the ankles of patients with CAI could be increased compared with those with healthy ankles after specific exercise training for mitigating CAI [49,51]. However, we are the first to further examine the ECC and CON of eversion and inversion strengths at various angular velocities and compare training and nontraining groups in the context of the ankles of patients with CAI. Research on this topic is rare, and comparing the effects of ankle eversion or inversion on isokinetic strength after a balance training program specific to patients with CAI remains challenging.

This study had several limitations. First, the participants with CAI self-reported their symptoms and ankle sprain history, which could be misleading when determining CAI status. Second, dorsiflexion and plantar flexion movements were not involved in the measurement of joint position sense and isokinetic strength. The anterior and posterior kinetic chain involving ankle stability was not discussed after the training programs. Third, lack of long-term follow-up could not confirm the cure of CAI or long-term effects. Four, our exercise training programs may be of insufficient duration and exercise implementation as normal fitness training. Our results revealed that there were very small or small effect sizes for CAI in dynamic balance, joint kinesthesia ability, and muscle strength of the ankle invertor and evertor after 6-week WBV or balance training. We researched the effects of the 20-min exercise comprised of three exercise movements performed on a balance ball or vibration platform. The variability of movement design may be inadequate, and the progress of training intensity maybe also insufficient, resulting in a very small or small effects on improvement of muscle strength, proprioception and balance ability. Investigation on the effects on different program designs of WBV or balance training in future studies is recommended.

## 5. Conclusions

Compared with the control group, female athletes who participated in 6-week exercise training programs incorporating a vibration platform and balance ball exhibited very small or small effect sizes for CAI in the SEBT, the joint position sense test, and the isokinetic strength test; in addition, COM and ECC at an ankle inversion of 30°/s were enhanced. We observed no differences among the variables within the two exercise training programs. A balance training program combining WBV training with a balance ball may be a potentially effective strategy for mitigating CAI, but further research is required to confirm these results.

## Figures and Tables

**Table 1:** Demographics of the participants.

	Group A (*n* = 21)	Group B (*n* = 21)	Group C (*n* = 21)	*p*
Age (y)	20.31 ± 1.28	20.43 ± 1.25	21.23 ± 1.47	0.08
Height (cm)	168.34 ± 5.78	166.8 ± 6.84	169.53 ± 4.78	0.32
Weight (kg)	61.01 ± 22.39	58.83 ± 13.14	58.67 ± 16.54	0.89
Dominant leg (Lt/Rt)	5/16	4/17	5/16	0.91
Composite score of CAIT	19.21 ± 1.89	19.14 ± 2.01	19.25 ± 1.91	0.89

Left, Lt; Right. Rt; Cumberland Ankle Instability Tool, CAIT.

**Table 2:** Outcome of ANOVA with the factors for the assessed variables.

Assessed Variables	Group			Time			Time × Group		
	F	*p*	ηp^2^	F	*p*	ηp^2^	F	*p*	ηp^2^
Composite of star excursion balance test	5.30	0.03	0.54	67.78	0.001	0.87	17.84	0.001	0.79
Active repositioning									
15° of ankle inversion	3.35	0.08	0.42	12.37	0.006	0.55	0.48	0.63	0.09
0° in ankle neutral position	6.59	0.01	0.59	12.26	0.006	0.54	1.58	0.25	0.26
10° of ankle eversion	0.51	0.61	0.10	8.27	0.01	0.45	1.60	0.25	0,27
Passive repositioning									
15° of ankle inversion	1.27	0.32	0.22	1.15	0.32	0.10	0.34	0.71	0.07
0° in ankle neutral position	0.16	0.84	0.03	0.67	0.42	0.06	0.18	0.83	0.03
10° of ankle eversion	0.56	0.58	0.11	0.70	0.41	0.06	0.87	0.44	0.16
Ankle inversion									
30°/s COM (N-m/kg)	3.01	0.10	0.40	0.04	0.82	0.01	4.49	0.04	0.50
30°/s ECC (N-m/kg)	14.02	0.002	0.75	5.56	0.04	0.35	0.71	0.51	0.13
120°/s COM (N-m/kg)	1.42	0.28	0.24	2.44	0.14	0.19	0.97	0.41	0.17
120°/s ECC (N-m/kg)	1.83	021	029	001	056	001	022	045	0.04
Ankle eversion									
30°/s COM (N-m/kg)	1.53	0.26	0.25	2.76	0.12	0.21	0.53	0.60	0.10
30°/s ECC (N-m/kg)	0.16	0.85	0.03	0.49	0.48	0.04	0.22	0.80	0.04
120°/s COM (N-m/kg)	2.73	0.11	0.37	4.12	0.07	0.45	4.07	0.06	0.47
120°/s ECC (N-m/kg)	0.34	0.71	0.07	4.72	0.06	0.32	3.22	0.09	0.41

COM: concentric contraction; ECC: eccentric contraction.

**Table 3:** The main outcome measurements in two training groups using linear regression analysis.

	Group A			Group B		
	B	95% CI	*p*	B	95% CI	*p*
Composite of star excursion balance test	2.52	−8.29–13.34	0.58	−2.92	−6.68–0.84	0.10
Active repositioning						
15° of ankle inversion	−0.05	−0.19–−0.09	0.03	0.01	−0.08–0.12	0.71
0° in ankle neutral position	0.30	0.04–0.56	0.02	−0.02	−0.13–−0.001	0.04
10° of ankle eversion	−0.05	−0.25–0.15	0.55	0.02	−0.04–0.10	0.38
Passive repositioning						
15° of ankle inversion	−0.15	−0.32–0.01	0.05	−0.02	−0.09–0.03	0.34
0° in ankle neutral position	−0.02	−0.15–0.11	0.69	0.02	−0.03–0.07	0.36
10° of ankle eversion	0.05	−0.09–0.21	0.41	−0.009	−0.07–0.05	0.73
Ankle inversion						
30°/s COM (N-m/kg)	−0.07	−0.15–−0.01	0.04	−0.01	−0.03–0.008	0.17
30°/s ECC (N-m/kg)	0.04	−0.14–0.23	0.56	−0.005	−0.05–0.04	0.80
120°/s COM (N-m/kg)	−0.03	−0.11–0.04	0.31	−0.006	−0.03–0.02	0.64
120°/s ECC (N-m/kg)	0.03	−0.09–0.16	0.53	0.002	−0.02–0.03	0.88
Ankle eversion						
30°/s COM (N-m/kg)	−0.02	−0.09–0.04	0.36	−0.02	−0.04–0.005	0.09
30°/s ECC (N-m/kg)	0.02	−0.07–0.12	0.60	−0.01	−0.04–0.02	0.48
120°/s COM (N-m/kg)	0.06	−0.06–0.18	0.26	−0.005	−0.03–0.02	0.69
120°/s ECC (N-m/kg)	−0.07	−0.25–0.10	0.34	−0.01	−0.04–0.008	0.13

COM: concentric contraction; ECC: eccentric contraction.

**Table 4:** Results of star excursion balance test in three group.

	Group A		Group B		Group C	
	Pre-	Post-	Pre-	Post-	Pre-	Post-
Anterior	80.63 ± 9.78	88.27 ± 10.83	76.16 ± 7.56	91.30 ± 4.94 ^+^	78.31 ± 12.40	81.45 ± 6.48
Anterolateral	72.35 ± 8.26	79.61 ± 15.14	71.74 ± 7.73	82.71 ± 8.54 ^+^	75.65 ± 5.36	72.93 ± 2.88
Anteromedial	87.01 ± 15.30	93.69 ± 9.58 *	85.18 ± 6.14	98.25 ± 7.07 ^+^	87.99 ± 11.85	80.63 ± 11.20
Posteromedial	81.27 ± 13.50	91.08 ± 13.51	85.59 ± 10.64	98.59 ± 11.04 ^+^	85.20 ± 9.91	84.71 ± 11.86
Posterior	71.62 ± 13.16	85.42 ± 16.69	70.49 ± 13.95	97.33 ± 12.54 ^+^	73.53 ± 10.34	79.50 ± 7.21
Posterolateral	68.10 ± 10.76	80.14 ± 12.39 *	63.37 ± 13.12	91.65 ± 13.01 ^+^	68.17 ± 13.02	69.68 ± 6.47
Medial	87.27 ± 16.96	93.62 ± 8.91	84.64 ± 6.90	97.73 ± 4.82 ^+^	86.84 ± 8.54	91.15 ± 5.10
Lateral	64.73 ± 13.36	70.33 ± 13.39 *	65.85 ± 13.26	85.58 ± 16.49 ^+^	67.41 ± 8.20	59.69 ± 3.06
Composite	76.62 ± 9.80	85.27 ± 11.76	75.38 ± 5.83	92.89 ± 7.80 ^+^	77.89 ± 4.60	77.47 ± 5.07

* *p* < 0.05, Group A vs. Group C, between-group using Bonferroni test; ^+^ *p* < 0.05, Group B vs. Group C, between-group using Bonferroni test.

**Table 5:** Results of joint position sense measurements in three group.

	Group A		Group B		Group C	
	Pre-	Post-	Pre-	Post-	Pre-	Post-
Active repositioning						
15° of ankle inversion	7.78 ± 5.60	4.67 ± 2.49 *	7.32 ± 3.67	5.14 ± 1.84 ^+^	8.33 ± 1.40	6.73 ± 1.66
0° in ankle neutral position	6.94 ± 3.83	4.55 ± 2.87 *	9.52 ± 2.71	6.55 ± 1.40 ^+^	9.06 ± 2.94	9.25 ± 1.02
10° of ankle eversion	7.22 ± 4.94	4.35 ± 2.24 *	7.65 ± 3.31	4.55 ± 2.16 ^+^	7.00 ± 4.10	6.84 ± 2.92
Passive repositioning						
15° of ankle inversion	6.17 ± 4.72	5.55 ± 3.45	6.45 ± 3.56	4.23 ± 1.07	7.67 ± 4.43	4.79 ± 2.51
0° in ankle neutral position	6.78 ± 3.32	5.21 ± 3.47	5.40 ± 3.03	5.16 ± 3.86	5.86 ± 4.31	5.16 ± 3.12
10° of ankle eversion	6.41 ± 3.42	4.50 ± 2.74	6.75 ± 2.69	6.25 ± 3.62	6.45 ± 4.36	6.70 ± 3.78

* *p* < 0.05, Group A vs. Group C, between-group using Bonferroni test; ^+^ *p* < 0.05, Group B vs. Group C, between-group using Bonferroni test.

**Table 6:** Results of isokinetic strength test in three groups.

	Group A		Group B		Group C	
	Pre-	Post-	Pre-	Post-	Pre-	Post-
Ankle inversion						
30°/s COM (N-m/kg)	28.47 ± 9.79	32.27 ± 9.25 *	27.52 ± 9.26	29.91 ± 6.80 ^+^	26.89 ± 11.14	21.90 ± 7.35
30°/s ECC (N-m/kg)	30.23 ± 6.53	33.15 ± 9.75 *	29.73 ± 5.61	32.45 ± 6.17 ^+^	23.06 ± 9.06	23.31 ± 7.13
120°/s COM (N-m/kg)	26.81 ± 7.14	32.00 ± 10.89	23.11 ± 11.33	26.44 ± 7.35	24.45 ± 12.83	24.35 ± 10.40
120°/s ECC (N-m/kg)	29.61 ± 11.18	31.75 ± 9.42	26.05 ± 8.83	23.96 ± 8.17	26.63 ± 11.22	27.27 ± 10.77
Ankle eversion						
30°/s COM (N-m/kg)	22.87 ± 5.36	26.74 ± 6.79	20.40 ± 8.85	21.39 ± 8.61	20.12 ± 4.00	21.61 ± 8.83
30°/s ECC (N-m/kg)	28.18 ± 6.09	30.83 ± 7.15	28.20 ± 8.24	29.73 ± 7.24	28.59 ± 7.99	27.74 ± 8.80
120°/s COM (N-m/kg)	24.84 ± 8.86	29.22 ± 8.18	21.00 ± 12.16	32.08 ± 3.48	21.08 ± 4.30	22.39 ± 4.91
120°/s ECC (N-m/kg)	29.65 ± 7.10	32.90 ± 8.28	26.72 ± 10.23	35.64 ± 5.74	29.87 ± 8.91	28.35 ± 7.10
Angular velocity (30°/s)						
COMeverton/COMinverton	0.85 ± 0.25	0.90 ± 0.36	0.76 ± 0.23	0.81 ± 0.45	0.84 ± 0.31	0.90 ± 0.43
ECCeverton/ECCinverton	0.97 ± 0.29	1.02 ± 0.43	0.96 ± 0.25	0.98 ± 0.35	0.95 ± 0.32	0.98 ± 0.48
ECCeverton/COMinverton	1.05 ± 0.30	1.07 ± 0.46	1.21 ± 0.47	1.06 ± 0.44	1.09 ± 0.64	1.23 ± 0.52
Angular velocity (120°/s)						
COMeverton/COMinverton	1.05 ± 0.46	1.05 ± 0.52	1.08 ± 0.49	1.01 ± 0.34	1.27 ± 0.29	1.04 ± 0.38
ECCeverton/ECCinverton	1.20 ± 0.72	1.13 ± 0.51	1.14 ± 0.55	1.21 ± 0.46	1.16 ± 0.33	1.11 ± 0.46
ECCevertor/COMinvertor	1.18 ± 0.43	1.15 ± 0.49	1.31 ± 0.67	1.22 ± 0.38	1.25 ± 0.39	1.20 ± 0.49

* *p* < 0.05, Group A vs. Group C, between-group using Bonferroni test; ^+^ *p* < 0.05, Group B vs. Group C, between-group using Bonferroni test; concentric contraction, CON; eccentric contraction, ECC.

## Data Availability

Data is contained within the article.

## Abbreviations

WBV	whole-body vibration
CAI	chronic ankle instability
SEBT	Star Excursion Balance Test
CON	concentric contraction
ECC	eccentric contraction
